# Beyond Pain Scales: A Critical Phenomenology of the Expression of Pain

**DOI:** 10.3389/fpain.2022.895443

**Published:** 2022-05-24

**Authors:** Nicole Miglio, Jessica Stanier

**Affiliations:** ^1^Philosophy Department, State University of Milan, Milan, Italy; ^2^Women's and Gender Studies Department, University of Haifa, Haifa, Israel; ^3^Wellcome Centre for Cultures and Environments of Health, Politics Department, College of Social Science and International Studies, University of Exeter, Exeter, United Kingdom

**Keywords:** phenomenology, pain, imagination, lived experience, lived body, critical phenomenology, expression, patient experience

## Abstract

In this paper, we discuss the qualitative dimension of painful experiences by exploring the role of imagination and metaphorical association in the conceptualization and expression of pain. We employ an engaged critical-phenomenological approach to offer original analysis influenced by the perspectives of people in pain. The paper is organized into three parts. Part 1 reviews literature on the expression of pain, its communication, and its reception—attending in particular to the emphasis on verbalizing pain in healthcare contexts. We here discuss benefits and limitations of standard methods aimed at facilitating the meaningful expression of pain (such as “pain scales”) from the perspectives of patients and practitioners, respectively. We suggest that these methods might be importantly complemented by facilitating creative expression of painful lived experiences with respect to personal lifeworlds. Part 2 deals with the role of imagination and metaphorical association in making sense of pain. We explore how imagination is a cognitive and affective mode of experiencing the world which plays a crucial role in determining how pain is experienced, as well as helping to make sense of pain figuratively in relation to the lifeworld. In Part 3, we draw from principles of engaged phenomenology to foreground case studies in which projects have been able facilitate the intersubjective expression of pain. These examples demonstrate the value of attending to the contours of painful lifeworlds in their specificity, affording both agency and accessibility in their communication, while remaining mindful of the complex power relations which govern perceived legitimacy and testimony relating to the transformation of pain. The overall paper aims to contribute to literature on qualitative pain research on both theoretical and practical levels. By exploring the expression of pain through phenomenology, we aim to enrich current debate on the qualitative experience of pain. We also seek to critically highlight the socio-political dimensions which frame painful experiences, their expression, their lived significance, and their treatment.

## Introduction

For decades, the International Association for the Study of Pain (IASP) defined pain as “[a]n unpleasant sensory and emotional experience associated with actual or potential tissue damage, or described in terms of such damage” (1). In an article that has sparked much important debate, however, Cohen, Quintner, and van Rysewyk proposed an alternative definition of pain from that of the IASP (1). These authors drew on the phenomenological experience of pain—as a portent of a threatening future, as a corporeal experience, as a source of meaning, as a threat to existential integrity, and as involving an intersubjective perspective—in order to “embrace pain as a shared phenomenon” [(1), p. 6]^1^. Their proposed alternative to the old IASP definition characterizes pain as “a mutually recognizable somatic experience that reflects a person's apprehension of threat to their bodily or existential integrity” [(1), p. 6]. The IASP definition has since shifted away from the implication that those in pain are able to describe it, to be better inclusive of those who are unable to articulate pain, such as infants and animals (“An unpleasant sensory and emotional experience associated with, or resembling that associated with, actual or potential tissue damage” [(2)]).

This debate concerning the intersubjective status of pain has been welcomed, even by those who remain unconvinced by alternative definitions, as “an inspiration for broadening our approach to pain assessment” [(3), p. 3]. As phenomenologists, we are greatly heartened by how these considerations have enriched contemporary debate in the clinical sphere. Having previously argued that it crucially matters how pain is constituted in experience within an intersubjective (social and political) context, and that this affects the very painfulness of a given experience (4, 5) we also greet this discussion concerning the shared dimensions of painful experience with enthusiasm. It is similarly heartening to see the biopsychosocial model of pain gaining traction in research and practice as a means to recognize how intersecting biological, psychological, and social determinants all contribute to the overall experience of pain (6, 7) and that none of these determinants taken in isolation will sufficiently capture painful experience in its complexity. We feel that this promising discussion of pain and its intersubjective dimensions calls for sophisticated critical-phenomenological analysis specifically concerning the *creative expression* of pain, which is an integral part of any pain assessment and subsequent sense-making of pain^2^^,^^3^. This is especially pressing since people experiencing pain—particularly in its chronic forms—stand to benefit from potential theoretical insights put into practice [cf. (8)]. Indeed, disabled activists, scholars, and communities have long called for a reevaluation of the treatment of pain and the uncritical equivocation between pain and broader structures that sustain suffering [(9), p. 203]. Intersubjective sense-making may not amount to straightforward healing or alleviation of pain, but it nonetheless involves an important transformation of relations that affords control and agency to those experiencing pain.

As phenomenologists, we want to remain faithful to this lived experience of complex communicability, expressivity, and amelioration regarding pain and its treatment. Phenomenology is a rigorous philosophical approach that explores how objects of experience present themselves and how they become meaningful [(10), p. 9]^4^. In this article, we draw especially from the critical-phenomenological approach, which “combines insights regarding embodied lived experience with analyses of socio-political structures and power relations which frame, inform, and shape that experience” [(11), p. 4]. We hope to introduce readers to key phenomenological concepts, alongside illustrative examples, with which to explore issues relating to the amelioration of painful experience. By attending to the role of imagination and metaphorical association in experiential constitution, from sensation to sociality, we offer a critical-phenomenological account that takes seriously the transformative potential of shared expression in this regard. Moreover, we draw from principles of engaged phenomenology to challenge “assumptions around narrativity and privileged articulacy,” to remain “mindful of how experience is lived through constellations of relations with others,” and to consider “the transformative potential of [people] sharing their experiences in meaningful ways, rather than merely assessing their ‘utility' in academic terms” (12). The case studies in this article are, along these lines, intended to illustrate the various ways in which pain can be transformed through intersubjective expression, while also attending to the complex power relations which govern perceived legitimacy and testimony relating to discussion of pain.

## On the Expression of Pain, Its Communication, and Its Reception

The sensations of my own body may be the only subject on which I am qualified to claim expertise. Sad and terrible, then, how little I know. “How do you feel?” the doctor asks, and I cannot answer. Not accurately. “Does this hurt?” he asks. Again, I'm not sure. “Do you have more or less pain than the last time I saw you?” Hard to say. I begin to lie to protect my reputation. I try to act certain [(13), p. 70].

It is often felt by people experiencing pain that they are expected, as patients, to offer linguistically articulate expressions of pain with purported medical utility; while the amended IASP definition goes some way to separating description and experience of pain, verbal expression is still a key part of clinical evaluation (e.g., rating pain on a scale of 1–10). In some academic contexts, by contrast, pain is assumed to be ineffable, intrinsically private, and impossible to communicate [(14), p. 53]. Both of these approaches to pain problematically obscure a more complex reality—a matter that significantly motivated Cohen et al. (1) in the formulation of their alternative definition. The creative expression of pain has a long history, and contemporary patient groups and disability activists have been able to facilitate profound communicability through poetry, imagery, and all manner of communal artistic expression (see Part 3). It is very much possible—and often vitally important in relations of care—to receive another person's expression of pain, to understand something of its painful nature, and to demonstrate this understanding. We can accept this philosophically without requiring an identity of experience between the person in pain and their companion(s) [(9), p. 206; (4), p. 106]. We know intuitively when other people “get it”. In this way, pain assessments are not merely instrumental means to understanding pain from a medical perspective; from a patient's perspective, this can be an important ethical interaction that can offer a sense of connection or alienation, depending on how the pain expressed has been received in this context.

Given that people are rarely offered other explicit opportunities to express their pain in the process of diagnosis and treatment, this encounter can take on additional and perhaps disproportionate significance. It is unclear that the assessment of pain can itself serve as a therapeutic intervention, and yet the responses of practitioners can here set the tone for how people relate to their pain moving forward. Without alternative avenues to explore shared understanding of pain, so much can depend upon this particular clinical encounter. This is not lost on practitioners, who are often acutely aware of how the multiple demands on the clinical encounter can compromise opportunities for compassionate care. As Disher (15) writes:

It is concerning to imagine to what degree we may be failing to help our patients by assuring them that concerning feelings are “normal” or by being unable to understand the experience they are describing. It is not uncommon to have a sense that something is being missed, and one wonders if a phenomenological toolkit that could be quickly at hand could be used in these moments to support assessment, diagnosis, and treatment [(15), p. 1,097].

The experience of shared understanding can have a hugely significant impact on the experience of pain itself (16). This shared understanding is often made possible by enabling people in pain to explore the shape of their pain and its impact with respect to their life as a whole—this concerns how the phenomenon of pain presents in the context of a particular lifeworld, to use a phenomenological term [cf. (17)]^5^. While the pain itself may linger, with no particular end in sight, a *transformation* of painful experience can be facilitated through meaningful intersubjective expression of pain, in which, as Hovey writes, “patients become people again” [(18), p. 12]. This can be the impetus for both attitudinal and material change in lifeworlds of people in pain. Painful sensations are put into relief by experiential circumstances which are not always inevitable but about which people can feel more or less hopeful depending on this perceived possibility of change:

Do I trust in any healthcare provision to which I have access? How long do I anticipate this pain will continue as a result, and does that anticipation feel bearable? Does this pain feel shameful, and do I feel worthy of care? These aspects of the painful experience may, in fact, problematically intensify or normalize these very pain sensations, depending on the intersubjective social and political context within which I find myself [(4), p. 109].

Opportunities to discuss pain this broadly are rare in clinical encounters, which are primarily focussed on curing the physical body of its malady. However, without a detailed sense of how pain manifests in the lifeworld, medical consultation can feel frustratingly generic and detached for patients [(19), p. 3]. Especially in cases of chronic pain, individual painful circumstances shape and color the lifeworld and, in “attending to these complexities of painful experience and associated suffering, a radically different notion of care may emerge as appropriate for each person beyond unsympathetic and clinical elimination of pain altogether” [(4), p. 111]—it is, after all, not always possible for treatment to offer a straightforward “cure,” and the unique significance of chronic pain for the patient must be taken seriously when exploring alternative treatment avenues. Ideally, treatment would respond specifically to the expression of pain for each individual in their particular situation.

So what is it like to have one's pain subject to the assessment of another? When I close my eyes, tense my body, and hold my breath when it hurts, try as I might not to flinch, what does this bodily expression tell you about my pain? What if I tell you it burns, or it feels sharp, or it aches? How can you know what this pain means to me? Given that painful sensations are only given directly in first-person experience, the attempt to gather up subjective pain into expression can feel particularly fraught. Indeed, as the IASP has come to recognize, not everyone experiencing pain is able to do this. Famously, in her seminal work *The Body in Pain*, Scarry argues that pain can “destroy language” [(14), p. 53]. Deep pain can indeed render linguistic expression, or even the attempt to conjure up words, void and impossible, such that a person in pain can but cry out in agony. It is interesting, however, that Scarry extrapolates from the impossibility of linguistic expression to claim that pain “brings with it all the solitude of absolute privacy with none of its safety, all the self-exposure of the utterly public with none of its possibility for camaraderie or shared experience” [(14), p. 53]. Certainly, the urgency and aversiveness of painful experiences can underscore the fundamental separation between first and third person perspectives; it is precisely when we most need someone to understand our pain, to care that it hurts us, and to know how to cure it that others “can turn away in disbelief and disregard,” since the pain *is* ultimately subjective [(4), p. 106]. To a certain extent, if someone tells you their pain is unbearable then you can but take their word for it (or choose to disbelieve); you cannot verify this claim with firsthand experiential evidence. However, we contend, unlike Scarry, that this appeal and desire for others to understand our pain actually discloses a very real possibility for the “camaraderie and shared experience” that she suggests pain might prevent.

Without discounting the plausibility of painful experiences in which people feel absolutely unreachable and inconsolable, we can, in fact, conceive of instances in which painful experiences are recognized and meaningfully understood by others without direct access to the painful sensations themselves. Such relations of empathy make it possible to offer care or cure, and these relations can develop between loved ones, between people who discover their respective pain bears resemblance, and importantly between patients and practitioners. Recognition that pain can indeed be shared in this intersubjective sense led Cohen, Quintner, and van Rysewyk to emphasize “mutual recognizability” in their alternative definition of pain [(1), p. 6]. They draw from Martin Buber's phenomenological analysis of first and third person perspectives, among other approaches, and conclude that the clinical encounter is, or should be,

a legitimate (socially sanctioned) and safe communal space in which both parties can accept and negotiate the meanings of the experience, including the testing of boundaries, thereby creating a therapeutic relationship [(1), p. 5].

As much as practitioners might aspire to facilitate such a space, this description is very much idealistic; in reality, imbalances of power, intercultural barriers, competing demands on time, negative prior experiences, pressure on resources, and other such factors can color the clinical encounter and prevent the negotiation of meaning in therapeutic terms (4).

It is important to note, given that the clinical encounter can be fraught, that individuals *are*, of course, capable of self-reflexively altering their experience of pain outside of medical spaces. This is not, however, the same as expecting individuals to simply “get on with it” alone; those looking to ameliorate pain must recognize that external support structures play a key role. This much is already recognized by most biopsychosocial conceptualizations of pain (6, 7). Isolation, loneliness, and rumination have also been linked to the exacerbation of the painfulness of experiences. These magnify the undesirability of the experience, and feelings of helplessness are core components of catastrophic thinking that can diminish recovery in the “functioning” of pain patients (20, 21). Positive interventions are most often facilitated within a fretwork of social relations, which more or less explicitly encourage and make possible the individual transformation of painful experience [cf. (22)]. It is crucially important that conceptions of pain do not treat people as solely and individually responsible for making sense of their painful experiences, since the circumstances that have enabled one person to address their own pain may not be afforded to other people. The clinical encounter thus garners much of its significance in the analysis of painful experience from the fact that it is the *common* space in which one seeks a medical explanation and assistance that cannot be found elsewhere (23). This does not mean that the encounter will necessarily represent the beginning and end of a person's understanding of their own pain. The encounter is, however, framed by these specific concerns, and the expression of pain is understood by each party in this interaction accordingly. For a medical practitioner, a meaningful expression of pain might importantly reveal details pertinent to a diagnosis or relevant for signposting a patient to better support. For a patient, as illustrated by Biss's account quoted at the beginning of Part 1, it can be unclear which expression of pain will be recognized and accepted as valid by others. Most (in)famously, pain scales have highlighted verbalized expression as key to medical understanding of pain.

Medical tools to assist the expression of pain proliferated in the twentieth Century, especially in the West after the Second World War, including three key models of pain measurement: “psychophysics, multidimensional questionnaires using standardized descriptors, and scales which rate the intensity of pain” [(24), p. 15]. In 1939, Dallenbach listed 44 words in total to, respectively, describe “the temporal course of pain, its spatial distribution, fusions or integration with pleasure, affective coloring, and qualitative attributes” [(24), p. 16–17]. The McGill Pain Questionnaire was developed in 1975 by Melzack, in consultation with panels of students, patients, and doctors, to identify words to describe sensory, affective, and evaluative dimensions of pain and to rank these words according to pain intensity (25, 26). Gracely and Dubner (27) sought clinical utility and accuracy in their proposed five properties of an ideal verbal pain measure, as well as seeking the possibility of absolute valid comparison of pain across groups. From pain charts (28) to descriptive terms (25) and visual-linguistic scales (29), these developments helped medical practitioners and researchers to recognize the utility and importance of subjective reports of pain, as well as furnishing them with means to record something resembling a pain “measurement.” Indeed, by the late 1990s, nursing literature began to refer to pain as “the fifth vital sign” (30). These notable and influential examples from twentieth century Western medicine, to mention just a few, illustrate how endeavors to facilitate the expression of pain have developed with respect to medical understanding.

While the medical profession now largely recognizes the importance of taking reports of pain into account, the communication of pain through scales and measures can be challenging for people experiencing the pain firsthand. Eula Biss (13) further describes the difficult process in her creative writing essay “The Pain Scale”:

Determining the intensity of my own pain is a blind calculation. On my first attempt, I assigned the value of ten to a theoretical experience—burning alive. Then I tried to determine what percentage of the pain of burning alive I was feeling.

I chose thirty percent—three. Which seemed, at the time, quite substantial.

Three. Mail remains unopened. Thoughts are rarely followed to their conclusions. Sitting still becomes unbearable after 1 h. Nausea sets in. Quiet desperation descends.

“Three is nothing,” my father tells me now. “Three is go home and take two aspirin.”

It would be helpful, I tell him, if that could be noted on the scale [(13), p. 72].

When the pain scale becomes the medium and vehicle for the expression of pain, “the questionnaire displaces the patient's own story, sidesteps the issue of pain's private meaning, and disrupts the potential for humane communication between patient and doctor” (31). The encounter *means* something crucially different to patients and practitioners, however much interpersonal relations of care mediate their communication, as Cohen et al. (1) might hope. As Padfield notes, “[b]y the time a patient ends up in a pain clinic there can be a wide gulf between the agendas of patient and clinician [...] and the significance it holds for them both. There is thus an urgency to find a means of crossing that gulf” [(32), p. 151].

Cohen et al. (1) do, however, offer a clue in their article as to how to realize a sense of intersubjective and mutual recognition of pain: “Through creative expression,” they say, “differences of point of view can be resolved and new possibilities are allowed to emerge” [(1), p. 5]. *Creative expression* is here upheld as key to facilitating understanding of pain between the person in pain and potential practitioners who might bear witness. But why restrict such expression to the consulting room and to the remit of the medical profession? What extraordinary transformations of pain might be rendered possible if practitioners could *signpost* to creative outlets, or if people could creatively express their pain in communities where they already feel at home? And finally, given that creativity is here seen as integral to the processing of pain, how can we address inequalities that can preclude people in pain from expressing themselves freely? As critical phenomenologist Cressida J. Heyes observes:

Ordinary life in the context of the pressures of postdisciplinary neoliberalism often feels compressed, demanding, teetering on the edge of possibility, utterly draining, yet also out-of-control, micromanaged by distant institutions and individuals. The response from even the most privileged individuals cannot always be to sit up, pay attention, work harder, work to change ourselves [... Sometimes] the only possibility of resistance (or even the only viable response) might be to detach from experience, to evade pain and fatigue, to slow down, and [...] to alter or even to lose consciousness [(33), p. 7].

For this reason, attempts to offer *generalizable* solutions or frameworks that might facilitate the expression of pain are unlikely to succeed in attending to the particular lifeworlds of people experiencing pain. General frameworks risk replicating social, political, and economic determinants of pain and will almost certainly limit the creative scope for people to explore their own painful experiences. A far more radical understanding of the role of imagination in the expression of pain can take us beyond the use of pain scales and toward a transformation of social and material conditions. To this end, we turn to critical phenomenology as an approach that “seeks not only to describe but also to repair the world” [(34), xiv].

## Phenomenology of Imagination and Expression

Phenomenology, as a philosophical approach, explicitly concerns itself with understanding and explicating processes of embodied meaning development. Phenomenology thus offers a way to address and understand pain as it is lived through and comes to bear meaning—an aspect of experience that is often overlooked by pathological or clinical accounts which emphasize dimensions of pain which are broadly quantifiable (9). It is vitally important to acknowledge that individuals experience and respond to pain differently within the same cultural contexts, and that pain is therefore not straightforwardly determined by external structural factors only [though these factors manifestly and importantly matter, as evidenced by critical-phenomenological analysis: see (4)]. While people become familiar with their pain through shared intersubjective environments and norms, their experience of pain develops and accrues distinctively within the particular context of their individual lifeworlds, and thus pain is constituted and embodied differently according to personal context and circumstance.

Phenomenology is sometimes characterized as the neutral description of the world as it is perceived from the first-person perspective, free of presuppositions and normative judgment; the approach is therefore sometimes criticized for taking individual experiences too seriously and for abstracting structural considerations out of the picture [(35); cf (36)]. There is, however, a promising thread within the phenomenological tradition that acknowledges how reflecting on experience can actually *open up* possibilities for affecting change (12, 37–40). This conception of phenomenology makes explicit the fact that “the phenomenologist renews their understanding of certain phenomena in the world—at a particular time and in a particular place—through the activity of critical reflection, and this reflection generates a new orientation and world-view with respect to the lifeworld” (12)^6^. The process of taking a reflective stance toward lived experiences can enable their *transformation* and *renewal—*phenomenology can help to illuminate the significance of objects in experience, explore which aspects of their significance are structural or contingent, and aid the expression of lived experience toward meaningful change [(40), p. 87]. This can be a particularly liberating avenue when some aspect of experience presents itself as especially urgent—perhaps, for example, during a painful experience.

The creative dimension underlying the transformation of sense is something that we hope to illuminate in this article, with a view to aiding practical understanding of imagination in the ameliorative expression of pain. Transforming how one understands and relates to one's pain—especially chronic pain—can have dramatic effects on everyday life. Having given an overview in Part 1 of the expression of pain in clinical assessment, we now delve into a phenomenological analysis of how imaginative expression enables people to make sense of their pain.

When experiencing pain, people are sometimes able to imaginatively shape their conception of their painful feelings to cope better with their impact (41–43). In this context, we argue that imagination should be conceived as a cognitive and affective strategy for transforming lived reality and making sense of personal experience. Traditional conceptions of imagination have, by contrast, upheld a distinction between body and mind, and have generally associated imagination solely with the “rational” side of the human being; as Irving (44) astutely points out, “it is curious [...] that the imagination is often seen as the faculty of fancy and a disengaged mind rather than as constitutive of bodily experience and practice” [(44), p. 298]. It is, indeed, strange to assume that imagination concerns only an abstract and disembodied “mind,” since the range of imaginative possibilities garner their meaningful significance precisely *through* the embodied entanglement of emotion, biology, and lived experience. On this matter, phenomenology is an approach that explicitly recognizes the character of the body as both a material object and a living organism. Throughout canonical phenomenology, the physical and psychical components of the self are not conceived as separate ontological entities, but are instead considered integral to understanding the body as a whole (45–47).

For the purposes of this article, we are interested in the following two aspects of the expression of pain: (a) how the imaginative process incorporates the experience of pain; and (b) how the expression of pain may be facilitated through metaphorical association. To these ends, we hold that imagination should be understood in relation to the “as-structure” within associative experience. Imagination is, in this sense, can be understood as a particular form of “quasi” perception, as Summa, Fuchs, and Vanzago explain with the following example:

If you try to imagine how it would be to meet a friend you haven't seen since your school time, you would somehow find yourself exploring that possibility: for instance, you would try to figure out how this person might look now after so many years, how s/he may have changed while still having some of the same bodily and/or expressive traits; also, you may imagine how it would feel like for you to have this person sitting nearby after so much time, etc. [(48), p. 6].

Imagination is thus better conceptualized as a form of “quasi” perception; in this example, my perception is overlaid *as if* my school friend were here now, even if I am fully aware that they are actually not here. The imaginative association is based on the as-structure, which Tengelyi similarly explores here, again from a phenomenological perspective:

Wherever something is taken as something (this as that), i.e., wherever something is in reality complex, manifold, disparate and even, upon individual consideration, is of a different kind than another, counts, from a certain point of view, as the same as the other (as being identical with the other), we may witness the emergence and the fixation of sense, making sense approachable, available, and even graspable [(49), p. 80].

This mechanism based on the as-structure thus reveals how consciousness receives, associates, or constitutes something within experience as meaningful in a given way. For example, I experience this pain *as* normal or familiar, *as* located in my head, *as* something to which I can attend in various ways—and this happens below the level of deliberate consciousness. While I can actively imagine what it might be like for my school friend to be here now, there is a more passive sense in which prior association through the as-structure renders the whole experience as intelligible and recognizable at all. Sense-making is, in this way, a complex process that involves multiple evolving as-structures. Moreover, certain especially critical moments can shatter prior understanding (and its related *as-structures*), demanding new conceptions in order for lived experience to make sense anew. Experiences of pain can comprise this kind of transformative event. Indeed, experiences of pain viscerally demonstrate that imagination is rarely a straightforwardly neutral mental exercise, but instead importantly involves an affective dimension:

[I]magining [...] involves something more like genuine rehearsal, “trying on” the point of view, trying to determine what it is like to inhabit it. It is something I may not be able to do if my heart is not in it. If we understood better why imagining [...] requires your heart to be in it, we would understand better what is being resisted when we resist [(50), p. 105].

We argue that imagination plays a crucial role in determining how the self makes sense of experiences of pain figuratively. Imagination is not only understood here as a cognitive tool, but also as a practical and situated way of dealing with painful experiences that is not always straightforwardly conscious. So what is special about imagination and why do we believe that focusing on imagination can inform pain treatment in a practical sense? First of all, it is crucial to bear in mind that pain is not a thing, a state, or a condition, but rather a “process that involves the whole person and whose complexity lies in the way it implicates all kinds of different biological structures and layers of meaning” [(51), p. 121]. Pain is therefore a phenomenon that cannot be categorized straightforwardly as a sensation or feeling, since it involves the totality of the human self (51, 52). The imaginative and figurative conception of pain can therefore be understood as an attempt to grasp this complex phenomenon through the as-structure. As Scarry points out, pain can be experienced *as if* it is caused by something coming from outside the body [(14), p. 12]. Ahmed (53) similarly grasps this aspect by pointing out that “we construct imaginary objects or weapons to take up” and with which to grasp experiences of pain [(53), p. 21]. We might imagine the intrusion of an external object into our bodily space and then seek to re-establish inner balance by addressing the implied cause of our pain. The absence of an actual object that causes the painful feeling is thus complemented by a metaphorical sense, and a related description, of some imagined material thing. In describing one's personal pain, it is not unusual to employ expressions such as “I feel pain *as if* there are needles on my skin” or “my stomach hurts *as if* it is burning”. All these linguistic expressions should not be understood only as idiosyncratic means to express pain, but rather as potential modes of *communication* with others. As Geary (54) shows, metaphors are employed creatively in expressing states that are resistant to expression, and pain is an especially notable form of experience which challenges linguistic communication. Indeed, metaphor can express a non-verbal as-structure conceived prior to the intersubjective encounter, but only rendered communicable in that instant (i.e., “I realize now that my pain has always felt like needles on my skin, though it has only just occurred to me to express it like that to you.”).

To explore this sense of the as-structure in another example, let us consider Ahmed's description of menstrual pain, in which she writes that,

In the example of period pain [...], I also create an imagined object. The pain is too familiar—I have felt it so many times before. I remember each time, anew. So I know it is my period, and the knowledge affects how it feels: it affects the pain. In this instance, the blood becomes the “object” that pushes against me, which presses against me, and that I imagine myself to be pushing out, as if it were an alien within [(55), p. 27].

Again, Ahmed points out that we *shape* the object of our pain by individuating a part of our own body as the cause of the painful sensation. By means of our imagination, we individuate, separate, and give a new form to our self-understanding of our own body, e.g., the blood in the example above. The bodily part, felt *as if* it was an external object, is then objectified as something potentially or actually harmful. Leder describes a similar sense of “sensory intensification” in which the painful area of the body “suddenly speaks up” thus interrupting and overwhelming experience—a hyper-presence he calls “dys-appearance” (56). In pain, we can experience body parts in their materiality, as physical objects that we recognize as our own but over which we have little control. Pain thus paradoxically makes us recognize our bodies as physical entities, and accordingly enhances a sense of alienation from our own corporeality. In attempting to make sense of this experience, we can resort to imaginative construction. As Grüny (51) describes, linguistic expression is a way of making sense of this alienation:

my abdominal pain feels as if I was being stabbed not just because this is my way of externalizing and objectifying a private experience, [...], but because I really do feel assaulted by an alien force that alienates part of my body, and I find myself nailed down without any way out [(51), p. 130].

These critical moments which give rise to the new formation of sense can be understood with reference to the as-structure. Alteration to the as-structure—when an experience previously received as “this” now makes sense also, or instead, as “that” —involves a breaking down of sedimented norms and expectations. This breakdown can also demand a revision of the relatively fixed narrative in which the original self-understanding was emplaced. Metaphor exemplifies the plasticity of the as-structure and its transformative potential; the creative reimagining of pain through metaphor makes possible new ways of being and relating. These considerations also lead us to recognize that painful experiences cannot be fully understood in quantitative terms. Although clinical practitioners sometimes need to grasp a patient's pain very quickly—calling for the use of a “pain scale” in some cases—in other circumstances (like chronic pain), the challenge is centered around managing pain through longer-term strategies in which the presence of personal pain is acknowledged and understood. In pursuit of this goal, the employment of imagination and metaphor offer potential both in a clinical context and for making sense of the broader lifeworld of the person in pain.

Practitioners might take up metaphorical language in order to make abstract medical knowledge accessible to patients (*practitioner to patient*); patients may want to express sensations, bodily feelings, and their psychical impact through metaphor to clinicians [*patient to practitioner*, see e.g., (57)] or to relate to others with similar experiences. This latter case (*patient to patient* or *person to person*) is of particular interest for the purposes of this article, because it captures the therapeutic potential of reaching an understanding with others in the mutual recognition of pain, as explored in Part 1. As Lakoff and Johnson explain in their seminal work *Metaphors we live by*, metaphor ought to be seen as “a matter of imaginative rationality” [(58), p. 325]. Metaphors make it possible to express and understand a given experience in terms of another through the as-structure by preserving coherence and mutual understanding. Far from being a mere rhetorical device, metaphor is not so much linked to language or intellect as with shared conceptual structures “including aspects of our sense experiences: color, shape, texture, sound, etc.” [(58), p. 235]. That metaphorical language deals with human experience in a holistic sense is an aspect of pain expression that we consider to be salient. In these respects, as phenomenologists, it is our primary occupation to recognize “the situated experience of the subject, not only in terms of ‘personhood' and abstract ‘rights' but, also and above all, as embodied and situated” [(4), p. 112].

## Collectively Making Sense of Painful Experiences

There is already widespread acknowledgment that imagination can be used to alter personal approaches to painful experiences. This kind of intervention effectively encourages the person in pain to find a way to express the pain to herself or perhaps to a practitioner who facilitates the intervention. Particularly in response to cases of chronic pain, pain without a known physical cause, or pain for which there is a long wait for treatment, practitioners have developed various frameworks and interventions through which patients are encouraged to renegotiate their attitude toward their pain to improve everyday life. Carel (8), for example, presents the idea of a phenomenological toolkit for patients—and, in fact, a whole facilitated workshop [cf. (15)]—as a means for people to attend to their relationship with their illness, to explore the ways it has changed their life, and to gain new understanding in light of these considerations.

The demonstrable benefits of these schemes are not in question here. As critical phenomenologists, however, we are interested in how shared understanding can facilitate transformation of painful experiences for the better—especially when the meaning is co-developed within a group dynamic of *shared power* to address marginalization. To this end, we are particularly curious about how endeavors *led by* people in pain have not only altered individual personal attitudes toward private pain, but also how they have transformed social relations in the medical contexts where they have taken place. These kinds of projects have the additional benefit, over more individualized approaches, of enabling horizontal cross-pollination of ideas across groups of patients, practitioners, communities, and others—developing networks of shared knowledge and more resilient means through which to transform the conditions which sustain or exacerbate painful experiences.

Approaches like this, which are able to foreground cultural contexts, importantly enable people experiencing pain to explore aspects of their intersubjective circumstances which exacerbate and sustain their painful experiences. As critical phenomenologists, we understand our experience “as emerging from structures of space, time, and embodiment; and always at the same time from contingent social and political structures that also constitute it” [(33), p. 134]; we therefore also understand cultural contexts as playing a crucial role in the constitution of painful experience and the lived possibility of its expression. The situated network of connections associated with a person in pain is highly pertinent to discussion of painful experience. From the perspective of someone experiencing pain, “[i]solation creates an additional layer of pain leading to depression, despondency, feelings of loss, purpose and value” [(18), p. 12]. As noted by Hodge, Itty, Samuel-Nakamura, and Cadogan, a pervasive sense that “we don't talk about it” (i.e., experiences of pain) can mean that “discussing such experiences can bring additional pain, suffering, and hardship to the family or community” [(59), p. 5]. This is a highly relevant consideration when it comes to addressing intersubjective contexts, which can serve to exacerbate or potentially ameliorate the lived significance of pain. Indeed, as Patsavas (9) notes, “when cultural discourses construct pain as the cause of feelings of devastation, they oversimplify complex cultural, historical, and political phenomena. More than that, they prevent us from examining the structural conditions that make experiences of chronic pain tragic” [(9), p. 204]—and these conditions are far from uniform across diverse contexts. Efforts which emphasize the specific meaning of painful experience for people in pain and which afford them agency in expression can, however, work toward mutual understanding and the transformation of painful experiences. When people experiencing pain are not simply framed as “patients” but instead as persons who have subjective interests, priorities, motivations, and capacities, holistic treatment of pain can facilitate and take seriously the importance of personal expression of pain with respect to their particular lifeworlds. Expression, in this context, must itself be imagined and regarded by people in pain *as* possible, *as* worthwhile, and *as* valued.

As we argued in Part 2, imagination plays a crucial role in the interpretation and reception of painful experiences. In this section, we explore some tangible examples that illustrate how people experiencing pain can find meaningful and transformative ways to share their perspectives (12). Shared endeavors enable participants to develop shared imaginaries through which they can make sense of their pain together. Painful experiences in these projects are not regarded as identical, nor are they presented as straightforwardly accessible to others. Nevertheless, projects like these proceed on the assumption that meaningful shared understanding of pain and its effects is worth pursuing—that coming together to understand painful experience is itself worthwhile and can affect change within contexts that “produce and sustain subjects in pain, as they are alternately marginalized, disbelieved, prioritized, or cared for” [(4), p. 102]. In this way, the conditions which shape experiences of pain can themselves be improved by those with firsthand knowledge of their effects. As phenomenologists, we are interested in how this important transformation of relations can afford control and agency to those experiencing pain, and specifically to those whose voices are otherwise marginalized. By introducing some key phenomenological concepts alongside illustrative examples, we hope to offer up a theoretical toolkit for pain researchers and clinicians with which to explore these issues. As such, we here review how different approaches can make it possible for individual experiences of pain to resonate through shared imaginaries and in the exploration of holistic treatment.

### Pain Cards

Quantitative and qualitative analyses have shown that the use of imagery in medical consultations improves the perceived quality of the session and encourages a more collaborative atmosphere in the consulting room (60, 61). Linguist Elena Semino shows that pain cards—a set of laminated images representing aspects of pain—can encourage patients to volunteer descriptions of their experience without solicitation by the practitioner, affording them more agency over the discussion topic and over the pain itself (61, 62). The images on the cards are open to interpretation. For example, one photo depicts a person's mouth closed with a clothes peg, while some others show a rag doll struggling to navigate a human-sized world [(63), p. 50–51]. There are 52 cards in total, offering up a variety of metaphorical images with which to express one's pain. After choosing a card, patients explain why and how they made this decision; this gives them the opportunity to reflect on which aspects of their pain they want to openly discuss. *Topic control*, as Semino reports [(61), p. 274], is an aspect of the practitioner-patient interaction that indicates power relations. For the patient in consultation, pain cards are tools that grant them epistemic agency; this shift can adjust the balance in power relations and give the patient the opportunity to use visual depiction of their pain to enhance linguistic expression:

The patient uses the image of the rag doll in the PAIN CARD as inspiration for describing herself via a simile (‘when I'm completely like a rag doll'). The following explanation clarifies the basis of the similarity: there are times when the person is so exhausted that, like a rag doll, she cannot walk. […] This patient uses a particular PAIN CARD as a springboard for a figurative description and a narrative that introduce three aspects of her life with chronic pain [(61), p. 281–282].

It is also significant that the use of pain cards does not so heavily rely on linguistic ability and instead relies on “visual imagination,” whereas pain scales are often less accessibly “designed for people who find adjectives and adverbs useful for them” [(60), p. 27]. So with the use of pain cards, patients with a range of linguistic backgrounds and abilities can regain a sense of agency in clinical encounters and can find figurative means to express how they feel. Indeed, Padfield et al. (64) argue “that exploring meaning is an essential part of understanding pain better, and that images introduced into an encounter become catalysts for both meaning-making and change” [(64), p. 80]—offering a practical means to enact a translation and transformation of the as-structure we described in Part 2.

More than this, however, as these authors suggest, a more radical sense of agency and connection is made possible where patients have designed the pain cards themselves:

[The pain cards] have been co-created with other pain patients and so could be seen as placing the bodies of other patients within the communication process. In another consultation for example, after using the cards, one patient says “At least I know I am not on my own” [(64), p. 78].

Though the authors only briefly remark on this mediated interaction between patients through the pain cards, the fact that other people in pain have created the figurative medium through which these interactions are facilitated is significant. No longer completely isolated, as Scarry (14) describes, in “all the solitude of absolute privacy with none of its safety, all the self-exposure of the utterly public with none of its possibility for camaraderie or shared experience” [(14), p. 53], the patient using pain cards is participating and reappropriating shared meaning in ways that can exceed the clinical encounter.

### Connections

Indeed, political agency, communal engagement, and social awareness can emerge in many ways from such projects—not so much to attest a given state of affairs regarding pain, but rather to express a point of view and related feelings. The *Face2face* project powerfully illustrates this point [cf (65, 66)]. Facilitated by Zakrzewska and Padfield, the project was aimed at improving dialogue in the consulting room (using pain cards) and supporting people in pain in the creative depiction of their own pain. Participants were invited to collaborate on the co-creation of “pain portraits.” Rather than “being an object on the other side of the lens” they used “objects, materials and the relations between them to evoke the internal abstract experience of pain, making it visible” and thus participants were “in charge of how [their pain] is seen by others” [(32), p. 155]. This resulted in the production of metaphorical images ranging from “exposed wires or rotting fruit” to more involved photographic experimentation [(32), p. 159]. The co-creation of pain portraits in this way made it possible for participants to regain some agency over their own pain and to challenge power dynamics in the medical setting. In fact, the process of creating a visual depiction of their pain not only helped participants to express their lived experience, but also initiated the renewal of sense-making with respect to their pain—a transformation of the as-structure. These types of interventions give people back the sense of control that long-term conditions can very often take away. Moreover, since these approaches do not treat participants as anonymous patients but instead respect the differences between people in pain and their individual situations, they can catalyze connection in something of a snowball effect. Aldous (63) participated in the *Face2face* project, for example, and describes how this “allowed me a chance to tell my story, to feel listened to and also to develop my own belief in my ability to identify triggers, reduce negative thoughts and improve my sleep” [(63), p. 52]. Consequently, as an occupational therapist, she was able to find ways to take up what she had learnt as a participant in her own practice with others:

I have been able to harness my own experiences and have used considerable effort and have used considerable effort to help others through their problems through creative participatory arts projects across our town. [...] I returned to work as occupational therapist [sic] with the eating disorders charity. I continued to incorporate the use of imagery alongside the cognitive behavior therapy protocol for eating disorders as a way of encouraging my clients to discuss their relationships with food and emotional states. This proved to be very powerful and not dissimilar to the way in which the PAIN CARDS are used in consultation.” [(63), p. 55].

Aldous' translation of her experience as a participant to her practice in her life and work more broadly exemplifies how people can share imaginative expression of pain across intersubjective contexts when health is conceived as part of networks of social relations. Agency and connection can be thus regarded as key aspects of how these alternative approaches to the expression of pain can transform the relations excluding or supporting people in pain.

### Zines

It may be more appropriate, in some cases, for the imaginative exploration and expression of pain to take place more concretely outside medical contexts. In many ways, decentring the clinical encounter can liberate the expression of pain from discussions that seek to “treat” or “cure” pain, and instead open up more creative and intersubjective avenues. *Ache Magazine* exemplifies this spirit, spanning the space between zines and magazines. Kirstie Millar, one of the editors, writes in the first issue about how *Ache* aims to bring together the voices of “self-identifying women and non-binary people,”

to explore, question and articulate our experiences with illness and pain. No illness is identical, our identities and our bodies unique [sic]. But through our collective and shared experiences, we can shift the conversation and be heard [(67), p. 4].

As an independent publication run by volunteers, *Ache* circulates poetry, literature, fiction, and visual art through which contributors and readers can explore their lived experiences. Poems in this first issue include “The Art of Blacking Out” by Annie Dawid, [(67), p. 6–7], “Prayer to Migraine” by Helena Hinn [(67), p. 20], and “Quantifiable” by Mel Reeve. The project demonstrates how the expression of pain can mediate both personal interests and the social spaces afforded to these people.

Zines have been used as an “alternative” means of sense-making for decades, sometimes explicitly in opposition to the medical setting, largely due to the accessibility of both their production and circulation. The Do-It-Yourself (DIY) ethos behind zines underpins their political potential, as summarized by Duncombe in *Notes from Underground: Zines and the Politics of Underground Culture* (68): “make your own culture and stop consuming that which is made for you” [(68), p. 7]. Holtzman et al. (69) notes how creating zines is relatively simple and affordable, since “all that is needed by an individual with a desire to express her/himself is access to a photocopier” [(69), p. 49]. Zines are made by juxtaposition and assembly of existing pre-constituted material. Zine creators are, in this way, able to refract their personal experiences as part of a wide intersubjective context. As Radway points out, they should not be read as idiosyncratic expressions of individuality:

I think zines should be read more for their radical generativity, for the way they combine and recombine rich repertoires of contradictory cultural fragments. They are experimental, multifarious performances, it seems to me, instantiations of multiple subject-positions [(70), p. 11].

Since zines are typically self-published, or published by small independent presses, they often circulate through localized networks and personal connections—through word-of-mouth and also, more recently, online. Access to zines is thus independent from institutional health settings, often deliberately so; zine production and dissemination instead relies on the self-organization of people who want to share their lived experiences in dialogical and creative ways. This can represent a powerful means for people to make sense of their experiences and make connections with others, sometimes in relation to experiences of illness and pain. Keyes, Peil, Williams, and Spiel, in their commentary on zines, note that,

Devalued identities are particularly susceptible to trauma by way of living their everyday lives in a system that overemphasizes minds over bodies, masculinity over femininity, whiteness over any other race, able-bodies over bodies rendered as socially disabled, and the like [(71), p. 24].

They grasp these dynamics by focusing on three elements encompassed by zines which are absent in contemporary health-care systems: “(1) reimagined possibilities, (2) flexible frameworks for empowerment and (3) community support” [(71), p. 22]. Zines responding to this context are often designed for and by people who have been historically marginalized by and discriminated against in health-care settings, as a way to share their stories, to find recognition, and to gain a sense of agency over their experiences.

The creation and distribution of zines are a powerful means of addressing personal experiences within more structural issues as concise, readily and inexpensively made, and easy to share. Moreover, their visual design can be tailored to suit “a spectrum of learning styles,” and can take into account additional accessibility requirements, such as the “translation into tactile imagery to complement expected Braille translations.” [(71), p. 24]. Zines thus have the potential to broker meaningful connections, encouraging “both inspiration and empowerment of producers as well as readers” [(69), p. 49]. The equivocal and multifarious creative possibilities afforded by zines also offer a unique space in which to explore the contours of an altered lifeworld.

### Community

Such explicitly arts-based projects may not be appropriate for everyone experiencing pain, however. At different times, and for different people, self-reflection on pain can be difficult and explicitly creative practices may not resonate. Where illness is associated with stigma, and when chronic pain is framed as a burden, people in pain may seek to avoid more direct or overt expression. Means of making sense of pain, in this context, may mean something altogether different. Researchers have explored the role of gendered social norms in experiencing and expressing pain, for example, revealing that men are less likely to seek social support and to share their painful experiences in certain socio-cultural contexts; gendered influences deeply impact how a person lives through and expresses their pain (72) and biases also affect pain assessment and treatment [e.g., (73, 74)]. The HOMEBASE project attends precisely to this context, as a “community-based project to reduce social isolation for men living with chronic pain” [(18), p. 13]. The project sets out “to offer every man suffering from chronic pain a community of care that extends beyond healthcare and into their communities to prevent social isolation and learn to live well with pain,” according to their own intuitive sense of how best to do so [(18), p. 13]. The project importantly recognizes that its accessibility depends upon attending to the specific needs of these men, and how they may wish, or not wish, to communicate about their pain. Hovey, as both researcher and participant in the project, describes the sense in which these men prefer “talking sideways”:

Men working together and talking sideways seems to be a preferred way of doing things. We want to get men together working on things and not address it through sharing your emotions, but coming together, becoming acquainted with each other, getting to know each other, trusting each other, and talking sideways (75).

Taking a more overtly emotional or creative approach would have run contrary to the inclinations of group members in this instance. Learning to live well with pain has, in this community context, involved recognition that a direct approach to addressing painful experiences may in fact make the project less accessible to those it would most benefit. The HOMEBASE approach involves connecting a man newly diagnosed with chronic pain with a buddy whose experienced perspective could “help them navigate the complexity of the pain world” (76). The contrast between making sense of pain with others and doing so alone can be stark:

When everything in our lives changes due to pain, when nothing is as it used to be (stuck in the liminal space) I feel exhausted, shattered, isolated and I do not know what to do anymore and I hide away from the world. Isolation creates an additional layer of pain leading to depression, despondency, feelings of loss, purpose and value [(18), p. 12].

In connecting with others through shared understanding of chronic pain, “there is transformation that occurs as patients become people again and are awakened by the multitude of challenges that lay ahead of them” [(18), p. 12]. The transformation that emerges here by no means replaces medical treatment of pain, but rather it is a transformation of lived sense. In this particular case, the as-structure is perhaps most importantly altered around perceptions of possibility:

As I recall my own reflections during this process although my levels of pain did not change, however, all other aspects of my life improved and continue to do so. The transformative possibilities keep unfolding [(18), p. 14].

## Conclusion

In this article, we have discussed how, while the clinical encounter is an event that can heavily influence the way people make sense of their pain, the process of making sense of chronic pain necessarily continues outside of and beyond medical settings. The clinical encounter is typically focussed on medical treatment, and therefore draws from expressive tools like pain scales which tend not to facilitate more holistic understanding and connection—this is, after all, not what they were designed to do. Appropriate means of expressing pain, as part of this sense-making process, thus vary accordingly.

We began Part 2 of this article with a quote from Biss's remarkable essay (13) on her experience of pain scales. As Jurecic (77) astutely commentates,

Biss's essay suggests many reasons why the numerical pain scale is an inadequate gauge of experience. As a writer, she finds greater resonance in the metaphors of the Beaufort scale. The highest number on that scale, which represents hurricane-strength winds, is described in a single word: “devastation.” When Biss was devastated by chronic pain, she recalls that she could ward off devastation by repeating and “secretly cherishing the phrase, “This too shall pass.” She found solace not in numbers or faith, but in words, rhythm, and ritual.

We have here attempted to explore how imaginative expression—through means such as “words, rhythm, and ritual” —can be meaningfully employed to transform painful experiences beyond pain scales in medical settings. In particular, we have emphasized the significance of expression in *intersubjective* contexts. Through our critical-phenomenological understanding of imagination and expression, we sought to show that holistic treatment of pain must begin by enabling the person in pain to express themselves with respect to their particular lifeworld. Through an analysis of the as-structure, we summarized phenomenological insights that arise when taking the roles of creativity and imagination in painful expression seriously, as well as their transformative potential. We then explored examples illustrating how communities of people experiencing pain, as well as practitioners, can make such imaginative transformations possible by engaging with the material and relational conditions affecting painful lifeworlds.

The examples discussed in this article may not be suitable for all people experiencing pain. Indeed, the point of affording people the opportunity to meaningfully participate in these projects is that they are able to voice their own perspective; the project should be tailored to the priorities of those participating, and therefore a top-down, one-size-fits-all approach would precisely miss the point. Some people may still prefer to make sense of their own pain within the context of their existing lifeworld and connections. Given the lack of literature emphasizing the importance of imaginative expression within intersubjective contexts, however, we hope that our phenomenological contribution in this article will shed light on a potential avenue for those seeking structural solutions to the amelioration of pain.

## Data Availability Statement

The original contributions presented in the study are included in the article/supplementary materials. Further inquiries can be directed to the corresponding author/s.

## Author Contributions

NM wrote Part 2, JS wrote Part 1, and both authors together wrote Part 3, the Introduction, and the Conclusion. All authors listed have made a substantial, direct, and intellectual contribution to the work and approved it for publication.

## Funding

This work was supported by the Israel Science Foundation [Grant No. 328/19], and the Wellcome Trust [Grant No. 203109/Z/16/Z].

## Conflict of Interest

The authors declare that the research was conducted in the absence of any commercial or financial relationships that could be construed as a potential conflict of interest.

## Publisher's Note

All claims expressed in this article are solely those of the authors and do not necessarily represent those of their affiliated organizations, or those of the publisher, the editors and the reviewers. Any product that may be evaluated in this article, or claim that may be made by its manufacturer, is not guaranteed or endorsed by the publisher.

## Footnotes

^1^ **Key definition: Intersubjectivity**
Intersubjectivity is the condition of being in the world alongside other subjects (i.e. other people). In the phenomenological tradition, the self is understood as intrinsically embodied and, by virtue of this embodiment, structurally related to other human beings—from basic interactions grounded in empathy to more complex social dimensions entailed by living together.

^2^**Key definition: Expression**. Expression can be understood broadly as acts which convey some subjective meaning—often, but not always, communicating that meaning to others. Expressive modes include speech, gesture, bodily movement, musicality, the creation of art, and many other acts. The received meaning of any given expression can ambiguously deviate from its intended meaning, depending on the context of its reception. Nonetheless, perhaps especially in creative acts of poetry or artwork, this ambiguity can lend itself to the overall expressivity of the act.

^3^ **Key definition: Sense-making**
In the context of phenomenology, sense-making is understood as the process by which individual and collective experiences become meaningful. While some aspects of sense-making take place on an unconscious level, being able to make sense of one's experiences consciously—in relation to objects, structures, and others in the world—enables one to act deliberately on the basis of lived experiences and to communicate it to others.

^4^ **Key definition: Phenomenology**
Phenomenology is a philosophical tradition stemming from the work of German philosopher Edmund Husserl at the beginning of the 20th Century. It has developed into various different philosophical legacies, of which Existentialism and Hermeneutics are among the most prominent. While there are various internal philosophical debates within the phenomenological field, its various interpreters generally all maintain that the first-person perspective is primary in the production of knowledge. Lived experience is understood as integral to the sense-making processes of the self.

^5^**Key definition: Lifeworld**. The lifeworld is the shared and communal world as experienced first-hand by the self in everyday life. Everyone has individual, perceptual, and social experiences that are foundational in the way they engage in the world thereafter. By emphasizing the importance of the lifeworld, phenomenology explores the experiential structures that underlie our immediate access to the world.

^6^**Key definition: Critical phenomenology**. This emerging theoretical and methodological approach investigates lived experiences by explicating the role of socio-cultural, political, economic, and historical dynamics. Although inherently diverse, critical-phenomenological approaches recognize that human experiences are shaped by factors like discriminatory practices, social injustice, and structural violence. By considering these elements of experience as “quasi-transcendental”, critical phenomenology proposes a notion of the self that is neither neutral nor abstract.

## References

[1] Cohen M Quintner J van Rysewyk S. Reconsidering the international association for the study of pain definition of pain. Pain Rep. (2018) 3:e634. 10.1097/PR9.0000000000000634 29756084 PMC5902253

[2] International Association for the Study of Pain. n.d. Terminology. Available online at: https://www.iasp-pain.org/resources/terminology/ (accessed January 24, 2022).

[3] Treede R. The International Association for the Study of Pain definition of pain: as valid in 2018 as in 1979, but in need of regularly updated footnotes. Pain Rep. (2018) 3:e643. 10.1097/PR9.0000000000000643 29756089 PMC5902252

[4] Stanier J Miglio N. Painful experience and constitution of the intersubjective self: a critical-phenomenological analysis. In: Ferrarello S, editor. Phenomenology of Bioethics: Technoethics and Lived-Experience. New York, NY: Springer (2021). p.101–14. 10.1007/978-3-030-65613-3_8 33909383

[5] Leder D. The phenomenology of healing: eight ways of dealing wth the ill and impaired body. J Med Philos. (2022) 47:137–54. 10.1093/jmp/jhab038 35137172

[6] Turk DC Okifuji AO. Psychological factors in chronic pain: evolution and revolution. J Consult Clin Psychol. (2002) 70:678–90. 10.1037/0022-006X.70.3.678 12090376

[7] Hadjistavropoulos T Craig KD Duck Cano A Goubert L Jackson PL. A biopsychosocial formulation of pain communication. Psychol Bull. (2011) 137:910–39. 10.1037/a0023876 21639605

[8] Carel H. Phenomenology of Illness. Oxford: Oxford University Press (2016). 10.1093/acprof:oso/9780199669653.001.0001

[9] Patsavas A. Recovering a cripistemology of pain: Leaky bodies, connective tissue, and feeling discourse. J Lit Cult Disabil Stud. (2014) 8:203–18. 10.3828/jlcds.2014.16

[10] Zahavi D. Phenomenology: The Basics. London: Routledge (2019). 10.4324/9781315441603

[11] Stanier J Miglio N Dolezal L. Pandemic Politics and Phenomenology - Editors' Introduction. Puncta. (2022) 5:1–12. 10.5399/PJCP.v5i1.1

[12] Stanier J. An introduction to engaged phenomenology. J Br Soc Phenomenol. forthcoming (2022). 10.1080/00071773.2022.2081533 PMC9255638 35813180

[13] Biss E. The pain scale. Creat Nonfiction. (2007) 32:65–84. Available online at: https://www.jstor.org/stable/44363570?seq=1

[14] Scarry E. The Body in Pain: The Making and Unmaking of the World. Oxford: Oxford University Press (1987).

[15] Disher T. Book review: phenomenology of illness by Havi Carel. J Evaluation Clinical Prac. (2017) 23:1096–8. 10.1111/jep.12812

[16] Boublil E. Compassion fatigue: assessing the psychological and moral boundaries of empathy. In: Ferrarello S, editor. Phenomenology of Bioethics: Technoethics and Lived-Experience. New York, NY: Springer (2021). p. 61–72. 10.1007/978-3-030-65613-3_5

[17] Dorfman E. History of the lifeworld: from Husserl To Merleau-Ponty. Philos Today. (2009) 53:294–303. 10.5840/philtoday200953317

[18] Hovey RB. The gift of pain with transformative possibilities. Patient Exp. (2018) 5:4:11–4. 10.35680/2372-0247.1248

[19] Hemingway A Norton L Aarts C. Principles of lifeworld led public health practice in the UK and Sweden: reducing health inequalities. Nurs Res Pract. (2015) 2015:124591. 10.1155/2015/124591 25642346 PMC4302377

[20] Drahovzal DN Stewart SH Sullivan MJ. Tendency to catastrophize somatic sensations: pain catastrophizing and anxiety sensitivity in predicting headache. Cogn Behav Ther. (2006) 35:226–35. 10.1080/16506070600898397 17189240

[21] Sullivan MJL Martel MO Tripp D Savard A Crombez G. The relation between catastrophizing and the communication of pain experience. Pain. (2006) 122:282–8. 10.1016/j.pain.2006.02.001 16545907

[22] Chambers C Craig KD Bennett SM. The impact of maternal behavior on children's pain experiences: an experimental analysis. J Pediatr Psychol. (2002) 27:293–301. 10.1093/jpepsy/27.3.293 11909936

[23] Rodemeyer L. I don't have the words. In: Mattens F, editor. Meaning and Language: Phenomenological Perspectives. Phaenomenologica. Dordrecht: Springer. (2008) 195–212. 10.1007/978-1-4020-8331-0_10

[24] Noble B Clark D Meldrum M ten Have H Seymour J Winslow M. The measurement of pain, 1945–2000. J Pain Symptom Manage. (2005) 29:14–21. 10.1016/j.jpainsymman.2004.08.007 15652435

[25] Melzack R. The McGill pain questionnaire: major properties and scoring methods. Pain. (1975) 1:277–99. 10.1016/0304-3959(75)90044-5 1235985

[26] Melzack R Torgerson WS. On the language of pain. Anethesiology. (1971) 34:50–9. 10.1097/00000542-197101000-00017 4924784

[27] Gracely RH Dubner R. Pain assessment in humans—a reply to Hall. Pain. (1981) 11:109–20. 10.1016/0304-3959(81)90144-5 7301399

[28] Hewer AJH Keele CA Keele KD Nathan PW. A clinical method of assessing analgesics. Lancet. (1949) 1:431–5. 10.1016/S0140-6736(49)90753-9 18112632

[29] Jensen MP Karoly P Braver S. The measurement of clinical pain intensity: a comparison of six methods. Pain. (1986) 27:117–26. 10.1016/0304-3959(86)90228-9 3785962

[30] Joel LA. The fifth vital sign: pain. Am J Nurs. (1999) 99:9. 10.1097/00000446-199902000-00002 10036564

[31] Lee C. Ann Jurecic – Illness as Narrative. (2015). Available online at: https://adogcalledpain.wordpress.com/2015/07/03/ann-jurecic-illness-as-narrative/ (accessed February 02, 2022).

[32] Padfield D. The photograph as a mediating space in clinical and creative encounters. In: Padfield D Zakrzewska JM, editors. Encountering Pain: Hearing, Seeing, Speaking. London: UCL Press (2021). p. 147–76. 10.14324/111.9781787352636

[33] Heyes CJ. Anaesthetics of Existence: Essays on Experience at the Edge. Durham: Duke University Press (2020). 10.1215/9781478009320

[34] Weiss G Murphy AV Salamon G. Introduction: transformative descriptions. In: Weiss G Murphy AV Salamon G, editors. 50 Concepts for a Critical Phenomenology. Evanston: Northwestern University Press (2020). p. 8–9. 10.2307/j.ctvmx3j22

[35] Scott JW. (1992) Experience. In: Butler J Scott JW, editors. Feminists Theorize the Political. London: Routledge (1992). p. 22–40.

[36] Stoller S. Phenomenology and the poststructural critique of experience. Int J Philos Stud. (2009) 17:707–37. 10.1080/09672550903301762

[37] Welton D. Structure and Genesis in Husserl's Phenomenology. In: Elliston F McCormick P, editors. Husserl: Expositions and Appraisals. London: Notre Dame Press (1977). p. 54–69.

[38] Toombs SK. The lived experience of disability. Hum Stud. (1995) 18:9–23. 10.1007/BF01322837

[39] Ahmed S. Queer phenomenology. Orientations, Objects, Others. Durham: Duke University Press. (2006).

[40] Steinbock AJ. Limit-Phenomena and Phenomenology in Husserl. Lanham: Rowman & Littlefield (2017).

[41] Peerdeman KJ van Laarhoven AM Peters ML Evers AWM. An integrative review of the influence of expectancies on pain. Front Psychol. (2016) 7:1270. 10.3389/fpsyg.2016.01270 27602013 PMC4993782

[42] Peerdeman KJ van Laarhoven AIM Keij SM Vase L Rovers MM Peters ML. Relieving patients' pain with expectation interventions: a meta-analysis. Pain. (2016) 157:1179–91. 10.1097/j.pain.0000000000000540 26945235

[43] Peerdeman KJ Geers AL Della Porta D Veldhuijzen DS Kirsch I. Underpredicting pain: an experimental investigation into the benefits and risks. Pain. (2021) 162:2024–35. 10.1097/j.pain.0000000000002199 33470747

[44] Irving A. The color of pain. Public Cult. (2009) 21:293–319. 10.1215/08992363-2008-030

[45] Husserl E. Ideas Pertaining to a Pure Phenomenology and to a Phenomenological Philosophy. First book: general introduction to a pure phenomenology. Kluwer (1982). 10.1007/978-94-009-7445-6

[46] Merleau-Ponty M. Phénoménologie de la Perception. Paris: Gallimard (1945).

[47] de Beauvoir S. Le Deuxième Sexe I. Les faits et les mythes. Paris: Gallimard (1949).

[48] Summa M Fuchs T Vanzago L. Imagination and Social Perspectives: Approaches From Phenomenology and Psychopathology. New York, NY: Routledge (2018). 10.4324/9781315411538

[49] Tengelyi L. The Wild Region in Life-history. Evanston: Northwestern University Press (2004). 10.2307/j.ctv47wftf

[50] Moran R. The expression of feeling in imagination. Philos Rev. (1994) 103:75–106. 10.2307/2185873

[51] Grüny C. No way out: a Phenomenology of pain. In: Dahl E Falke C Eriksen TE, editors. Phenomenology of the Broken Body. New York, NY: Routledge (2019). p. 119–136. 10.4324/9780429462542-8

[52] Geniusas S. The Phenomenology of Pain. Athens: Ohio University Press (2020). 10.2307/j.ctv224twdv

[53] Ahmed S. The contingency of pain. Parallax. (2002) 8:17–34. 10.1080/13534640110119597

[54] Geary J. I Is an Other: The Secret Life of Metaphor and How It Shapes the Way We See the World. New York, NY: Harper Collins (2011).

[55] Ahmed S. The Cultural Politics of Emotion. 2nd Edn. Edinburgh: Edinburgh University Press (2014).

[56] Leder D. The Absent Body. Chicago: University of Chicago Press (1990).

[57] Stewart M Ryan SJ. Do metaphors have therapeutic value for people in pain? A systematic review. Pain Rehabil J Physiother Pain Assoc. (2020) 10–23. Available online at: https://research.brighton.ac.uk/en/publications/do-metaphors-have-therapeutic-value-for-people-in-pain-a-systemat

[58] Lakoff G Johnson M. Metaphors We Live By. Chicago: University of Chicago Press (1980).

[59] Hodge FS Itty TL Samuel-Nakamura C Cadogan M. We don't talk about it: cancer pain and American Indian survivors. Cancers. (2020) 12:1932. 10.3390/cancers12071932 32708860 PMC7409157

[60] Padfield D Fitzgerald M. Introduction II: What is pain? A neurobiological perspective. In: Padfield D Zakrzewska JM, editors. Encountering Pain: Hearing, Seeing, Speaking. London: UCL Press (2021). p.17–32.

[61] Semino E (2021). Language and images in pain consultations. In: Padfield D Zakrzewska JM, editors. Encountering Pain: Hearing, Seeing, Speaking. London: UCL Press (2021). p. 269–85.

[62] Semino E. Descriptions of pain, metaphor, and embodied simulation. Metaphor Symb. (2010) 25:205–26. 10.1080/10926488.2010.510926

[63] Aldous L. Three testimonies from those living with pain. facial pain: managing facial pain through creativity. In: Padfield D Zakrzewska JM, editors. Encountering Pain: Hearing, Seeing, Speaking. London: UCL Press. (2021) p. 46–56.

[64] Padfield D Omand H Semino E Williams ACC Zakrzewska JM. Images as catalysts for meaning-making in medical pain encounters: a multidisciplinary analysis. Med Humanit. (2018) 44:74–81. 10.1136/medhum-2017-011415 29895594 PMC6031279

[65] Picturing Health. About. (2020). Available online at: https://www.picturing-health.com/about (accessed January 24, 2022).

[66] Picturing Health. Media Education. (2020). Available online at: https://www.picturing-health.com/about (accessed January 24, 2022).

[67] Millar K Reynolds-Grey R Millar K. Ache Issue No. 1 (2018) Independently published.

[68] Duncombe SR. Notes From the Underground: Zines and the Politics of Underground Culture. London: Verso Books (1997).

[69] Holtzman B Hughes C Van Meter K. Do it yourself… and the movement beyond capitalism. In: Graeber D Biddle E, editors. Constituent Imagination: Militant Investigations//Collective Theorization. Oakland: AK Press. (2007). p. 44–61.

[70] Radway J. Girls, Zines, and the Miscellaneous Production of Subjectivity in an Age of Unceasing Circulation. Speaker Series No. 18, Center for Interdisciplinary Studies of Writing and the Literacy & Rhetorical Studies Minor (2001).

[71] Keyes O Peil B Williams RM Spiel K. Reimagining (Women's) Health: HCI, Gender and Essentialised Embodiment. ACM Transactions on Computer-Human Interaction. (2020) 27:1–42. 10.1145/3404218

[72] McClelland LE McCubbin JA. Social influence and pain response in women and men. J Behav Med. (2008) 31:413–20. 10.1007/s10865-008-9163-6 18587638

[73] FitzGerald C Hurst S. Implicit bias in healthcare professionals: a systematic review. BMC Med Ethics. (2017) 18:19. 10.1186/s12910-017-0179-8 28249596 PMC5333436

[74] Samulowitz A Gremyr I Eriksson E Hensing G. “Brave Men” and “Emotional Women”: a theory-guided literature review on gender bias in health care and gendered norms towards patients with chronic pain. Pain Res Manage. (2018) 2018:6358624. 10.1155/2018/6358624 29682130 PMC5845507

[75] Concordia University. Homebase: A Community of Care for Men Enduring Pain and Isolation. Available online at: https://www.concordia.ca/research/perform/about/blog/20171121-Homebase.html (accessed January 24, 2022).

[76] McGill University Faculty of Dentistry. Homebase an Initiative to Address Chronic Pain and Prevent Social Isolation Among Men. (2016) Available online at: www.youtube.com/watch?v=B_aDp5yFZhs (accessed January 24, 2022).

[77] Jurecic A. A literary pain scale. Lancet. (2018) 391:111. 10.1016/S0140-6736(18)30022-9 29353610

